# Bifunctional Phosphorus Dendrimers and Their Properties

**DOI:** 10.3390/molecules21040538

**Published:** 2016-04-23

**Authors:** Anne-Marie Caminade, Jean-Pierre Majoral

**Affiliations:** 1 Laboratoire de Chimie de Coordination (LCC), CNRS, 205 Route de Narbonne, BP 44099, F-31077 Toulouse Cedex 4, France; majoral@lcc-toulouse.fr; 2 UPS, INPT, Université de Toulouse, F-31077 Toulouse Cedex 4, France

**Keywords:** dendrimers, dendrons, synthesis, phosphorus, fluorescence, catalysis, nanomaterials, biology

## Abstract

Dendrimers are hyperbranched and monodisperse macromolecules, generally considered as a special class of polymers, but synthesized step-by-step. Most dendrimers have a uniform structure, with a single type of terminal function. However, it is often desirable to have at least two different functional groups. This review will discuss the case of bifunctional phosphorus-containing dendrimers, and the consequences for their properties. Besides the terminal functions, dendritic structures may have also a function at the core, or linked off-center to the core, or at the core of dendrons (dendritic wedges). Association of two dendrons having different terminal functions leads to Janus dendrimers (two faces). The internal structure can also possess functional groups on one layer, or linked to one layer, or on several layers. Finally, there are several ways to have two types of terminal functions, besides the case of Janus dendrimers: either each terminal function bears two functions sequentially, or two different functions are linked to each terminal branching point. Examples of each type of structure will be given in this review, as well as practical uses of such sophisticated structures in the fields of fluorescence, catalysis, nanomaterials and biology.

## 1. Introduction

**Scheme 1:**
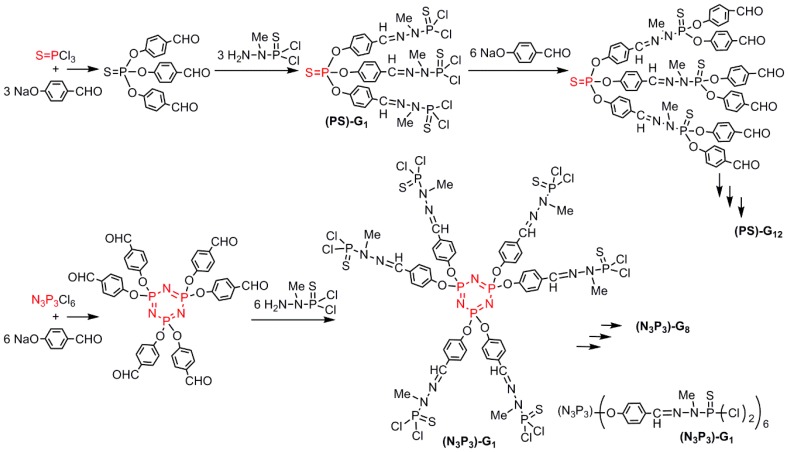
Divergent synthesis of phosphorhydrazone dendrimers, from two different cores. Linear representation for (N_3_P_3_)-G_1_ that will be used in most of the following figures.

Dendrimers are hyperbranched and monodisperse macromolecules, constituted of monomers associated radially from a central core in an iterative fashion. Due to their repetitive structure, dendrimers can be considered as a special class of polymers, but contrarily to polymers, they are never synthesized by polymerization reactions, but step-by-step. Each time a new layer of branching points is created, a new generation is obtained. High generations have been obtained in some cases: generation 9 for polyphenylene dendrimers [1], generation 10 for PAMAM dendrimers [2] and for polylysine dendrimers [3], generation 12 for phosphorhydrazone dendrimers [4], and recently generation 13 for triazine dendrimers [5]. In most cases, the internal structure has no specific function usable for further functionalization, and all the terminal functions are identical. However, it is often desirable to have two (or more) types of functions present in a single dendrimer. Some examples of such multifunctional dendrimers have been displayed in a recent review, essentially centered on organic dendrimers [6]. In the present review, we will emphasize the multiple possibilities offered by “inorganic” dendrimers (*i.e*., with inorganic elements at each branching point) [7], especially phosphorus-containing dendrimers, for the synthesis of diverse types of bifunctional dendritic structures. As shown in Scheme 1, the synthesis of phosphorus-containing dendrimers, mainly of the phosphorhydrazone type, necessitates two steps for each layer, starting from a central core which is generally either P(S)Cl_3_ [8] or N_3_P_3_Cl_6_ [9].

**Figure 1:**
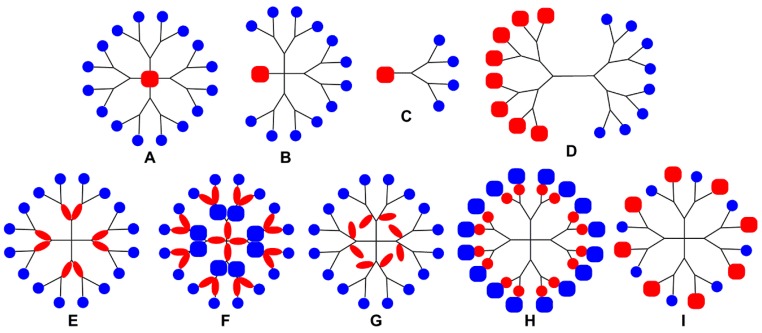
Types of bi-functional dendrimers. (**A**–**C**) one function at the core different from the terminal functions; (**E**–**G**) functions inside the structure, at one or several layers, different from the terminal functions; **D**, **H**, and **I**: two types of terminal functions.

This method of synthesis allows many modifications, leading to dendritic structures having at least two different types of functions. Indeed, besides the terminal functions, dendritic structures may have also a function at the core (A), or linked off-center to the core (B), or at the core of dendrons (dendritic wedges) (C). Association of two dendrons having different terminal functions leads to Janus (two faced) dendrimers (D). The internal structure can also include functional groups either on one layer (E), or linked to one layer (F), or on several layers (G). Finally, there are several ways to have two types of terminal functions, besides the case of Janus dendrimers D: either each terminal function bears two functions sequentially (H), or two different functions are linked to each terminal branching point (I) (Figure 1). Examples of each type of structure will be given in this review, as well as practical uses of such sophisticated structures.

## 2. Dendritic Structures with a Function at the Level of the Core

We will consider in this section only the cases for which the core affords a specific property. Structures of type A, B, and C (Figure 1) will be gathered in this section.

### 2.1. Functional Cores of Dendrimers (Type A)

**Figure 2:**
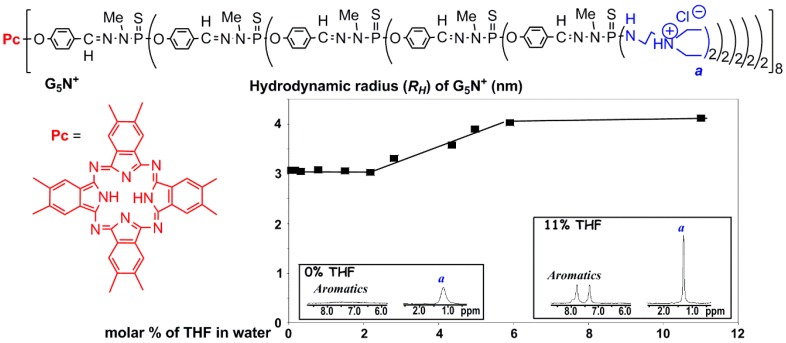
Generation 5 phosphorus dendrimer built from a phthalocyanine core and ended by ammonium groups. ^1^H-NMR spectra in water or water/THF mixtures, and variation of the hydrodynamic radius measured by diffusion NMR.

In this case, the core is the starting point of the synthesis, applying the method shown in Scheme 1. Thus, the core should have either P-Cl functions or aldehydes to continue the growing of the structure of phosphorus dendrimers. A phthalocyanine functionalized by eight aldehydes has been used as core for the synthesis of phosphorhydrazone dendrimers, using the process shown in Scheme 1. The process was carried out up to generation 5 from a free phthalocyanine, and up to generation 8 starting from its cobalt complex [10]. The phthalocyanine core was used for analyzing the properties of the internal structure and for determining the influence of each structural part (surface, branches, core) upon the whole structure, behaving as a sensitive sensor. There was no evidence for aggregation, except for generation 0, showing the effective isolation of the core starting from the first generation. Furthermore, both a hyperchromic and bathochromic effect on the Q-bands of the phthalocyanine with increasing generation was observed in the UV-visible spectra. Such behavior is indicative of an increased isolation of the chromophore, surrounded by the dendritic shell, which mimics a highly polar solvent. The fluorescence quantum yield increased with increasing generations. It was found also that the phthalocyanine core behaves as a highly sensitive optical sensor for H_3_O^+^ and OH^−^ [11]. The hydrodynamic radius (Rh) of generation 4 with aldehyde terminal groups and of generation 5 with ammonium terminal groups was measured by diffusion NMR (pulse gradient spin-echo) experiments. Rh decreased from 35.4 Å at 4 mol % of water to 32.5 Å at 64 mol % of water in THF for G_4_CHO, and from 41 Å at 90 mol % of water to 30 Å at 100 mol % of water for G_5_N^+^ [12], showing in both cases the hydrophobic nature of these dendrimers. Furthermore, ^1^H-NMR spectra carried out in pure water displayed only the signals corresponding to the ammonium terminal groups, but in water/THF mixtures, the signals corresponding to the internal structure were recovered (Figure 2). The generations 0, 1 and 4 were also studied by IR and Raman spectroscopies [13]. DFT calculations carried out on generations 0 and 1, afforded information in perfect agreement with the experimental data [14].

Some biological properties of the dendrimer in Figure 2 were studied. Cytotoxicity towards HeLa, HEK 293, and HUVEC cells was measured in standard MTT assays. The cytotoxicity was lower than that of lipofectin. This dendrimer was then used in transfection experiments in the presence of serum. It efficiently delivered a fluorescein-labelled oligodeoxyribonucleotide and a DNA plasmid containing the functional gene of EGFP (green fluorescent protein) into HeLa cells [15], showing that phosphorus dendrimers constitute a class of versatile transfection agents [16,17].

**Figure 3:**
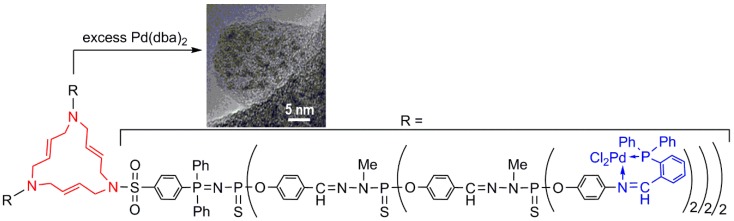
Dendrimer having complexes of P,N ligands and PdCl_2_ as terminal functions and a triazatriolefinic macrocycle as core. Addition of Pd(dba)_2_ induces the formation of Pd nanoparticles.

Another type of macrocyclic core, a triazatriolefinic macrocycle, was used as starting point for the synthesis of phosphorus dendrimers. This core has three triphenylphosphino groups, which reacted via Staudinger reaction with a phosphorus derivative possessing one azide and two aldehydes, affording P=N-P=S linkages. The properties of these linkages will be displayed later (see Section 3.2 and Section 3.3). The growing of the dendrimers was pursued from these aldehydes. The surface was functionalized either with the same macrocycle [18], or with P,N ligands able to complex palladium (II). In the latter case, the core was suitable for the complexation of Pd(0), but adding an excess (10 equiv.) of Pd(dba)_2_ (dba = dibenzylidene acetone) induced the formation of Pd nanoparticles (Figure 3) [19], suitable for catalysis experiments [20]. It was shown previously that the same type of macrocycles grafted to the surface of phosphorus dendrimers afforded Pt nanoparticles in the reaction with Pt_2_(dba)_3_. Furthermore, these Pt nanoparticles were organized in dendritic networks, whose size depended on the generation of the dendrimers [21].

**Figure 4:**
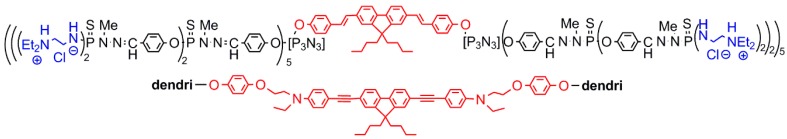
Two dendrimers with a TPA fluorophore as core have been used for imaging the vascular network of a rat olfactory bulb (**top**), and the blood vessels of a tadpole (**bottom**).

Other types of functional cores are fluorescent. Two-photon absorption (TPA) has attracted a lot of interest, owing to its applications in various fields, in particular for high-resolution three-dimensional fluorescent imaging of biological systems. Especially engineered organic chromophores having high TPA properties were used as cores of phosphorus dendrimers. The first step for the growing of dendrimers was the specific reaction of both phenol groups of the chromophore with two equivalents of N_3_P_3_Cl_6_, which afforded 10 Cl suitable for pursuing the synthesis. The last step was the peripheral functionalization with either a second type of fluorophore [22], or ammonium groups for inducing solubility in water. In the latter case, it was shown that the dendritic branches created a local setting that is favorable to TPA [23]. This type of dendrimer (Figure 4) was used for imaging *in vivo* the vascular network of a rat olfactory bulb [24], and the blood vessels of a tadpole [25].

A last example concerns an ammonium group used as core of phosphorus dendrimers having carboxylic acids as terminal functions. The reaction with carteolol, an anti-hypertensive drug for the treatment of glaucoma, led to a dendritic salt, which was solubilized in water and dropped in the eyes of rabbits. No irritation was observed, and the quantity of carteolol measured in the aqueous humor was increased compared to neat carteolol, presumably thanks to an increased time of residence of carteolol in the eye when interacting with the dendrimer [26].

### 2.2. Off-Center Functions Linked to the Core of Dendrimers (Type B)

The phosphorus dendrimers of type B in Figure 1 having an off-center function linked to the core were all obtained thanks to the specific reactivity of the hexachlorocyclotriphosphazene [27]. Indeed, it is possible to differentiate the reactivity of one Cl among six. The first examples in the field of dendrimers concerned compounds having one function of the carboxylic acid or hydrazone type linked to the core, with several Boc-protected amines as terminal functions, or one aldehyde or one ammonium linked to the core, with several fluorescent dansyl groups as terminal functions [28]. The fluorescence of the latter compound in dioxane/water mixtures displayed an unexpected behavior. Increasing the quantity of water up to a molar fraction of 0.8 in water induced an increased red-shift of the fluorescence wavelength from 492 to 524 nm. A dramatic blue-shift was then observed up to 501 nm, as the quantity of water was increased up to pure water. This phenomenon has to be related to differences in the viscosity of dioxane/water mixtures, which is maximal when the molar fraction is 0.8. This compound can thus be considered as a new sensing element for viscosity [29].

**Figure 5:**
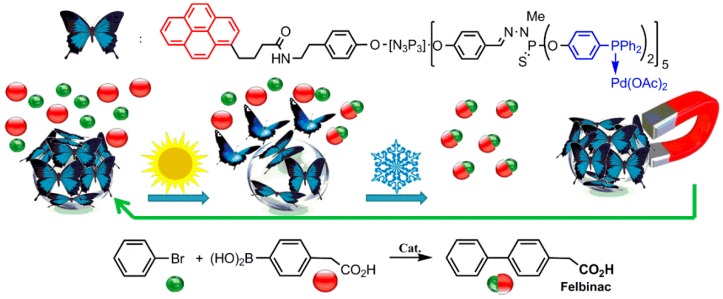
Dendrimer having a pyrene linked off-center to the core and palladium complex of phosphines as terminal groups. Illustration of the interaction of the pyrene at room temperature with the graphene layer covering cobalt nanoparticles. The dendrimers/Co nanoparticles are easily recovered using a magnet, to be reused in a new catalytic cycle. Illustration for the synthesis of felbinac.

Other bifunctional dendrimers constituted of one protected amine linked to the core, and either five phosphines or 10 phosphine imine ligands as terminal groups, have been also synthesized [30]. Having a pyrene linked to the core instead of the protected amine induced the possible interaction of the dendrimer with graphene layers covering cobalt nanoparticles. The complexation of the phosphine terminal groups with Pd(OAc)_2_ afforded a highly reusable catalyst for Suzuki couplings. Indeed, at room temperature, the dendrimer is stacked on the nanoparticle, whereas by heating it goes inside solution for performing the catalysis. By cooling to room temperature after the end of the catalysis, the dendrimers stacked again on the nanoparticle, so they can be easily recovered using a magnet and used again in the next run. This process has been successfully applied to the synthesis of felbinac (an anti-inflammatory drug) in quantitative yield, even after 12 re-uses [31]. The concept is illustrated in Figure 5. A recent review has gathered many other examples of phosphorus dendrimers used as catalysts [32].

**Figure 6:**
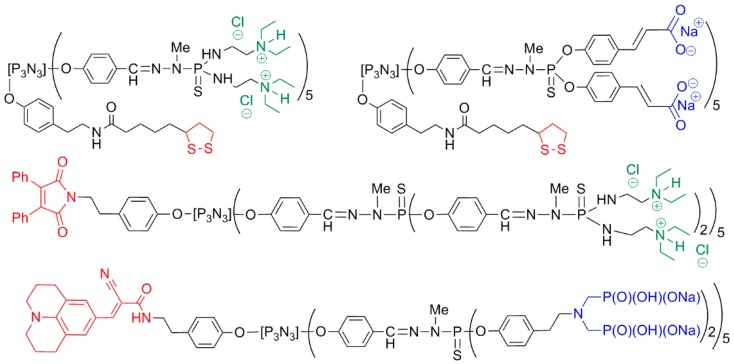
Examples of dendrimers with an off-center function linked to the core; **top**: dithiolane for the grafting to gold surfaces; middle: fluorescent maleimide group; **bottom**: fluorescent julolidine group.

Having one function emanating from the core different from all the other functions is also a useful concept in the field of materials. Dendrimers with a triethoxysilyl group linked to the core and Boc-protected amines were synthesized for the functionalization of porous silica [33] in view of trapping CO_2_ after deprotection of the amines. Also related to materials, but with an application in biology, dendrimers with a dithiolane group linked to the core and either positively charged ammoniums or negatively charged carboxylates as terminal groups were grafted to gold surfaces via the dithiolane function (Figure 6). The functionalized surfaces were used for cultures of human osteoblasts (responsible for the building of bones). The positively charged surfaces induced the death of osteoblasts, whereas the negatively charged surfaces induced the multiplication of the osteoblast [34]. However, only small dendrimers were usable for such purpose, as even the second generation could not be grafted, due to the burying of the off-center function inside the structure, which became non-accessible.

The same concept applied for studying biological properties was used in particular for grafting a fluorophore to the core. A maleimide derivative was linked either on all the terminal functions, or as a single copy to the core. Study of the fluorescence properties of both series of dendrimers in organic solvents shows that the grafting of the fluorophore directly to the core afforded highly fluorescent compounds [35]. In view of these properties, the second generation dendrimer with the maleimide linked to the core, was functionalized by ammonium terminal groups, to ensure the solubility in water (Figure 6). This dendrimer was brightly fluorescent in CH_2_Cl_2_, but deceptively poorly fluorescent in water, due to quenching induced by water. The cytotoxicity of this compound was relatively low towards two cancerous cell strains, HeLa and A549 cells, and surprisingly less toxic after 48 h than after 24 h. Association of this dendrimer with plasmid DNA analyzed by circular dichroism (CD) induced a disturbance of the helical B-type structure of DNA. In view of these results, this dendritic tool is potentially interesting for transfection experiments, but the low fluorescence intensity indicates that it is not suitable for monitoring the biological events associated with transfection [36]. A julolidine fluorescent group was also grafted off-center, and the surface of the first generation dendrimer was functionalized with azabisphosphonic salts (Figure 6). This compound was used to interact with purified human monocytes. Confocal microscopy demonstrated that this dendrimer penetrated inside monocytes very rapidly (within one minute) [37]. The analogous compound with a maleimide group instead of the julolidine group was also synthesized [38]. These compounds were synthesized among a large library of dendrimers ended by azabisphosphonic salts [39], which have numerous properties, for the multiplication of human natural killer (NK) cells [40], and for the potential treatment of rheumatoid arthritis [41] and multiple sclerosis [42]. In general, multicharged dendrimers, which are soluble in water, have many biological properties [43].

### 2.3. Dendrons (Type C)

Phosphorus-containing dendrons are all synthesized by divergent processes, contrarily to the first types of organic dendrons, which were synthesized by a convergent process [44]. In addition to dendrimers of type C, some of these phosphorus-containing dendrons have been used for the synthesis of more sophisticated architectures, in particular of type D (Figure 1, Section 2.4), but not only those. As it is not trivial to have a function at the core which will not react during the synthetic process, but which can be activated later, many studies have been carried out with the aim of exploring the diverse possibilities. The functions linked to the core and compatible with the growing of the dendrons were an allyl, a pyridine, an alkyl chloride or azide [45], a diethylphosphonate [46], a polyethyleneglycol (PEG) derivative [47], a phenol protected by a methyl ester, which can be deprotected [48], and an activated vinyl group able to react with various primary and secondary amines [49], compatible with phosphine surface groups [50]. The thermal stability of several dendrons of various generations with the activated vinyl group as core and diverse types of terminal functions (aldehyde, tertiary amine, nitrile) was measured. The lowest mass lost was for the second generation ended by aldehydes, lower than for the corresponding dendrimer built from the N_3_P_3_ core, presumably thanks to a polymerization of the vinyl group [51].

**Figure 7:**
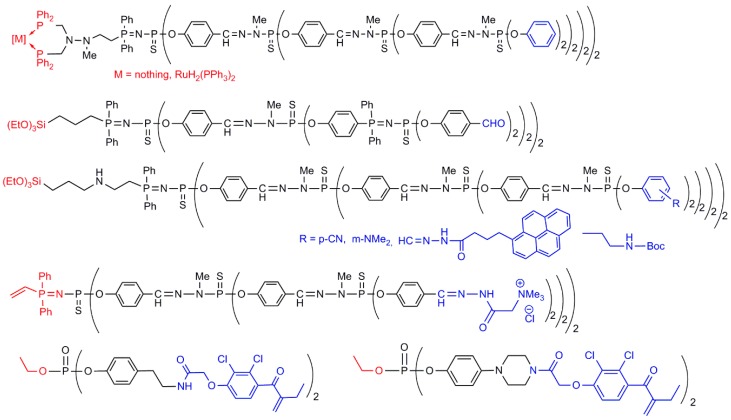
Examples of dendrons synthesized for applied purposes. From **top to bottom**: dendron for catalysis, Et_3_Si group as core for the grafting to silica, vinyl group for the grafting to polystyrene nanoparticles, and two small dendrons with two ethacrynic derivatives as terminal functions, tested as anti-cancer agents.

Several dendrons were also elaborated for diverse applied purposes. A dendron with a diphosphosphine as core, suitable for the complexation of ruthenium (Figure 7), was used as catalyst for diastereoselective Michael additions. The percentage of reaction, the diastereoisomeric ratio, and the possibility to recover and reuse the dendron were all comparable to those of a dendrimer having the same complex as terminal groups [52]. Several dendrons with a hydrolysable triethoxysilyl group as core and aldehydes, nitriles, secondary amines, or pyrene as terminal functions (Figure 7) were used in co-hydrolysis and polycondensations with TEOS (tetraethoxysilane), for the synthesis of dendrons/silica xerogels, via sol-gel processes. The dendrons were not damaged in the process, and were covalently grafted to silica [53]. The same family of dendrons, but having Boc-protected amines as terminal groups, were also synthesized recently [33]. 

Dendrons with an activated vinyl group at the core and Girard-T reagents (acethydrazide trimethylammonium chloride) as terminal groups (Figure 7) were used for their covalent grafting to polystyrene nanoparticles functionalized by cyclam. This simple and straightforward reaction gave rise to translucent aqueous suspensions of polystyrene nanoparticles, with an average size diameter of 16 nm. The polycationic dendritic shell provided a remarkable improvement of the colloidal stability. Indeed, the suspensions of nanoparticles covered by the dendrons were stable in the absence of surfactant, had a good tolerance to an increase of the ionic strength, could be redispersed in water after drying, and formed translucent hydrogels after one week at room temperature, all things impossible to carry out with the “nude” nanoparticles [54]. Hydrogels were previously obtained with water-soluble dendrimers decorated with Girard reagents as terminal groups [55]. Finally, very small dendrons having an ethoxy group as core and ethacrynic acid derivatives as terminal groups (Figure 7) were synthesized, together with various dendrimers also functionalized with ethacrynic acid, for testing their anti-proliferative activities against solid and liquid tumor cell lines. The dendrons displayed good anti-proliferative activities and a good “safety ratio” [56]. Dendrons can be grafted to various multifunctional cores to afford dendrimers. The most original core is certainly an oligophosphazene having different functional groups on each phosphazene unit, including two aldehydes. A dendron issued from the addition of methyl hydrazine to an activated vinyl core was reacted with the aldehyde functions, affording an unusual type of dendrimeric species, with a multifunctionalized oligophosphazene core and eight trifluoromethyl terminal groups [57]. Another dendron with a methylhydrazine core was used as topological amplifier. It was grafted to a tetraphosphorus macrocycle bearing four aldehyde groups. The steric hindrance induced by the dendrons allowed for the first time to detect topological differences in the macrocycle, which became detectable by ^31^P-NMR spectroscopy [58].

### 2.4. Dendrons for the Synthesis of Non-Symmetrical Dendrimers (Type D)

**Figure 8:**
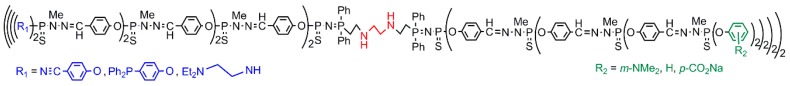
Examples of Janus dendrimers obtained by the association of two dendrons with a diamine.

A review from our group has gathered all the early examples concerning the use of dendrons for the synthesis of highly sophisticated dendritic architectures [59], thus only the most salient examples will be given here. Janus dendrimers (type D in Figure 1) are constituted of two dendrimeric wedges linked by their core, and having different terminal functions [60]. In the case of phosphorus-containing dendrimers, the first example was elaborated with dendrons up to generation 3 bearing an activated vinyl group at the core, and different terminal functions. One of the dendrons was reacted with a diamine by Michael-like reactions, affording a dendron with a primary amine at the core, suitable for the reaction with another dendron having an activated vinyl group as core, and different terminal functions. Various combinations of terminal functions were obtained in this way, such as nitrile and tertiary amine [61], triphenyl phosphine (or its oxide) and phenyl [62], or tertiary amine and carboxylate of different generations [63] (Figure 8). In the latter case, the Janus dendrimers issued from the coupling of two generation 3 dendrons were deposited on a silica surface pre-coated with 3-aminopropyl dimethylethoxysilane (3-APDMES). The Janus dendrimer strongly interacted with the positively charged silica surface through the carboxylate side, affording a neutral surface of amines. The amines were quaternized with methyl iodide, then a second layer of the same Janus dendrimers was added. Alternating alkylation of amines and deposit of another layer of Janus dendrimers was carried out up to the deposit of four layers. Such method using a single type of dendritic structure for layer-by-layer (LbL) deposit is an alternative to the deposit of negatively and positively charged symmetrical dendrimers [64], which has been applied in particular: (i) to silica cylindrical nanopores [65,66] for obtaining nanotubes of dendrimers [67], eventually accommodating quantum dots [68,69]; (ii) to microspheres for obtaining microcapsules of dendrimers [70]; (iii) to surfaces for the culture of cells [71]; and (iv) for elaboration of ultrasensitive (10^−18^ M) biosensors [72]. Associating the concept of dendrimers and nanotubes has let to fruitful properties, not only with phosphorus dendrimers [73].

**Figure 9:**
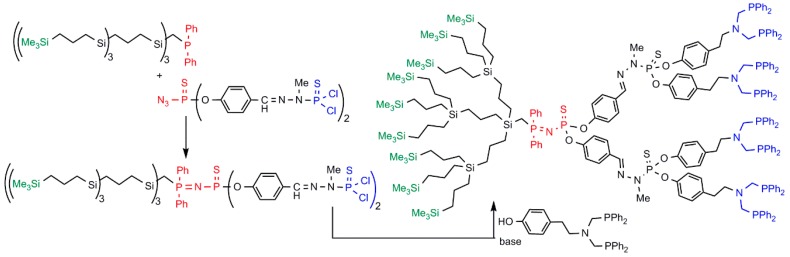
Synthesis of a Janus dendrimer by “click” Staudinger reaction, then functionalization on one side.

Besides the sequential use of a diamine for linking two different dendrons via Michael additions, a few additional methods have been used. They comprise the condensation reaction between a small dendron having an aldehyde at the core and fluorescent dansyl terminal groups, with another small dendron having a hydrazine at the core and Boc-protected tyramine as terminal groups. These protected groups could be deprotected by trifluoroacetic acid (TFA) to afford a water-soluble and fluorescent Janus dendrimer. An analogous compound was obtained by peptide coupling between a fluorescent dendron having a primary amine at the core and another dendron having a carboxylic acid at the core and Boc-protected tyramine as terminal groups [28]. The Staudinger reaction between azide and phosphine was also used for coupling two dendritic wedges having a very different structure. On one side a carbosilane dendron with a phosphine at the core, and on the other side a phosphorhydrazone dendron with a thiophosphoryl azide at the core and P(S)Cl_2_ terminal functions were readily associated. The Staudinger “click” reaction afforded a new type of Janus dendrimers, in which not only the terminal groups but also the full structure of the dendrons are entirely different. Furthermore, the P(S)Cl_2_ terminal function can be used for further functionalizations of the Janus dendrimer. Aldehydes, triphenyl phosphines, and azabisdiphosphines have been used to functionalize these Janus dendrimers [74] (Figure 9).

As shown in the previous example, Janus dendrimers can be modified after their synthesis, depending on the type of their terminal functions. For instance, when R_1_ in Figure 8 is a derivative of triphenylphosphine, it is able to react via Staudinger reaction with a dendron having an azide as core and nitrile [60], or NMe_2_ [61] as terminal functions, affording another type of Janus dendrimer, constituted of wedges of different size. A small Janus dendrimer was synthesized with four phenyl groups on one side and four aldehydes on the other side; the aldehydes were then used for the growing of dendritic branches, up to the third generation. The final compound is a Janus dendrimer having 16 aldehydes on one side, and four phenyl groups on the other side [75].

## 3. Dendritic Structures with Functional Internal Branches

The paramount importance of the internal structure of dendrimers, in particular for the biological properties, has been recently emphasized [39]. Thus, the presence of functional groups inside the structure is of special interest.

### 3.1. Functions as Constituents of the Branches at One or Several Layers (Case E)

**Figure 10:**
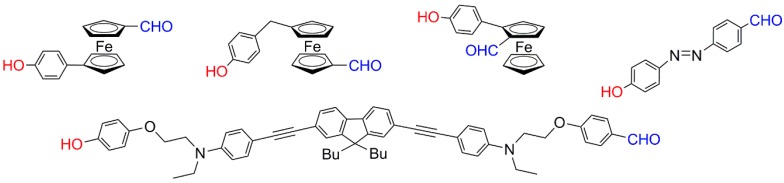
Examples of functional groups used at one or several layers inside the structure of phosphorhydrazone dendrimers, in replacement of 4-hydroxybenzaldehyde.

The easiest way to have specific functions inside phosphorhydrazone dendrimers consists in replacing 4-hydroxybenzaldehyde at one or several layers by a compound possessing both a phenol and an aldehyde. Several ferrocenes have been functionalized to fulfill these criteria (Figure 10). The first one has an aldehyde directly linked to one cyclopentadienyl ring (Cp) and a phenol linked to the other Cp. This ferrocene was used at all layers (three layers) of a phosphorhydrazone dendrimer. The ferrocenes in the inner layers are oxidized at the same potential, but the ferrocenes at the outer layer need a higher potential to be oxidized, due to the presence of the electron-withdrawing aldehyde [76]. An analogous ferrocene having a CH_2_ linker between the phenol and the Cp was introduced at the second level of third generation of dendrimers ended by ammoniums or carboxylates [77]. Planar chirality of ferrocenes is achieved by introducing two different groups on a single Cp in a controlled manner. Such type of ferrocene bearing a phenol and an aldehyde on a single Cp was introduced at a single level of phosphorhydrazone dendrimers (at generation 3, 5, or 9) then the growth of the dendrimer was continued for two generations, affording dendrimers of generations 5, 7 and 11, respectively. The influence of the “burying” of the chiral ferrocene upon the electrochemical and chiroptical properties was measured. It was shown that the chiroptical properties are not sensitive to where the chiral units are positioned. On the contrary, the electrochemical properties are very sensitive to the burying of the ferrocene, and more sensitive for the largest dendrimers [78].

Another function that could be sensitive to the burying inside the structure of dendrimers is the azobenzene, which generally undergoes an easy trans/cis isomerization upon irradiation, and thermal cis/trans isomerization. An azobenzene functionalized by OH on one side and an aldehyde on the other side (Figure 10) was precisely placed at one or several layers inside the structure of phosphorhydrazone dendrimers, having either aldehyde or P(S)Cl_2_ terminal functions. The position of the azobenzene groups had no influence on the absorption properties, and the value of the molar absorption coefficient (ε_max_) increased with the number of azobenzene groups in a simple additive way. On the contrary, the isomerization properties were highly dependent on the location of the azobenzene groups. Progressive burying inside the dendrimers led to a progressive reluctance to isomerize upon irradiation [79]. The isomerization has been also monitored by a gradient hydrogen-nitrogen multiple quantum coherence ^1^H-^15^N technique with natural abundance ^15^N [80].

Symmetrical fluorophores suitably engineered for TPA have been used as cores of dendrimers (see Figure 4). Unsymmetrical fluorophores functionalized with one phenol and one aldehyde are usable as branches of dendrimers (Figure 10). The fluorophore was grafted to the N_3_P_3_ core, and the surface was functionalized with an analogous fluorophore, affording a dendrimer with two layers of fluorophores. However, gathering a large number of fluorophores in a confined space induced a decrease of the fluorescence properties [25]. The same fluorophore was also incorporated at the level of the first generation, for the synthesis of biocompatible dendritic fluorophores, ended either by tertiary ammoniums (third generation) or by PEGs (second generation). Both dendrimers exhibited excellent one- and two-photon brightness, and are promising fluorescent labels for one- and two-photon fluorescence imaging bioapplications [81].

**Figure 11:**
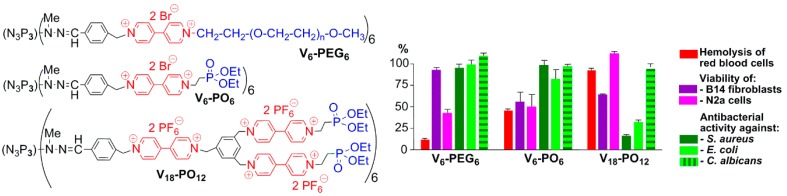
Examples of dendrimers incorporating viologen units in their structure, and some of their biological properties.

We have already seen many examples in which positive charges constitute the terminal groups of dendrimers (see in particular Figure 2, Figure 4, Figure 6 and Figure 7), but it is also possible to have positive charges inside the structure of dendrimers. For this purpose, viologen groups (doubly alkylated 4,4-bipyridine units) have been included in various dendritic structures, at one or two layers. Three representative examples among dozens [82] are shown in Figure 11. All these compounds possess biological properties, which have been extensively studied. First, interaction of these viologen dendrimers with human serum albumin (HSA) was examined. Some of them did not influence the albumin circular dichroism (CD) spectra, while others, in particular those having aldehyde terminal groups, quenched HSA fluorescence and changed the secondary structure of albumin [83]. Cytotoxicity, hemotoxicity, antimicrobial and antifungal activity of these dendrimers were also examined. Several conclusions could be inferred: (i) dendrimers bearing the highest number of viologen units cause more hemolysis; (ii) a few dendrimers, in particular the PEGylated one (V_6_-PEG_6_ in Figure 11), are not toxic toward B14 (non-cancerous) cells but are very cytotoxic toward N2a cancerous cells; (iii) most tested dendrimers exhibited good antimicrobial properties toward the Gram-positive strain *S. aureus*; (iv) some dendrimers bearing the highest number of viologen units (such as V_18_-PO_12_ in Figure 11) also limit the growth of Gram-negative strains *E. coli* and *P. vulgaris* [84].

The same series of dendrimers incorporating viologen units in their structure was also tested towards two enzymes: acetylcholinesterase and butyrylcholinesterase. These viologen-phosphorus dendrimers inhibited the activities of both cholinesterases, showing their potential as new drugs for treating neurodegenerative diseases [85]. This work was continued with the study of the influence of some of these dendrimers on cells of the central nervous system. The tested dendrimers (such as V_6_-PO_6_ in Figure 11) did not cause a strong cellular response, and induced a low level of apoptosis of murine hippocampal cell line. Treatment by these dendrimers induced a slight decrease of ROS level (reactive oxygen species) compared to untreated cells, in contrast with the increase of ROS species observed with PAMAM dendrimers [86,87]. Such decrease of ROS is correlated with a slight increase of catalase activity [88]. Other tests were carried out with embryonic mouse hippocampal cells, in particular to determine if the dendrimers could prevent damage of these cells caused by rotenone, which is a pesticide, insecticide, and a nonselective piscicide. All dendrimers reduced cell death compared with untreated controls. A lower level of ROS and a favorable effect on mitochondrial system were also observed [89].

**Figure 12:**
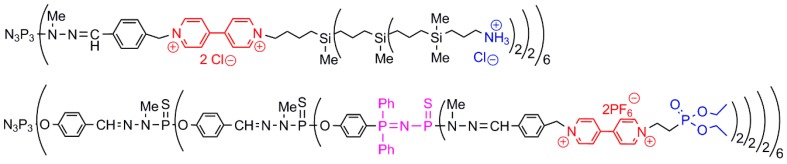
Examples of “onion peel” phosphorus dendrimers incorporating viologen units.

Other types of dendrimers incorporating viologen units in their structure have been recently obtained, as new examples of the synthetic “onion peel” [90] approach, *i.e.*, dendritic compounds having different types of layers. For this purpose, dendrimers functionalized with viologen units at the first layer were reacted with carbosilane dendrons of the first or second generation. The presence of primary ammonium groups as terminal functions (Figure 12) ensured the solubility in water. The biological properties of this new family of viologen/carbosilane dendrimers were tested. Most properties, including the toxicity, were related to the number of positive charges on the terminal functions, in particular towards the eukaryotic cells Chinese hamster fibroblasts (B14 cell line) [91]. Another family of “onion peel” dendrimers incorporating several types of functions in their structure have been recently synthesized. An example is shown in Figure 12, in which seven different types of phosphorus units are incorporated. This compound has phosphonate terminal groups, viologen units close to the terminal functions, and P=N-P=S linkages at one level inside the structure [92]. The role played by such linkage in the synthesis of layered dendrimers as well as its particular reactivity will be emphasized in the next paragraphs (Section 3.2 and Section 3.3).

### 3.2. Functions as Intrinsic Constituents of the Branches of Layered Dendrimers (Case F)

**Figure 13:**
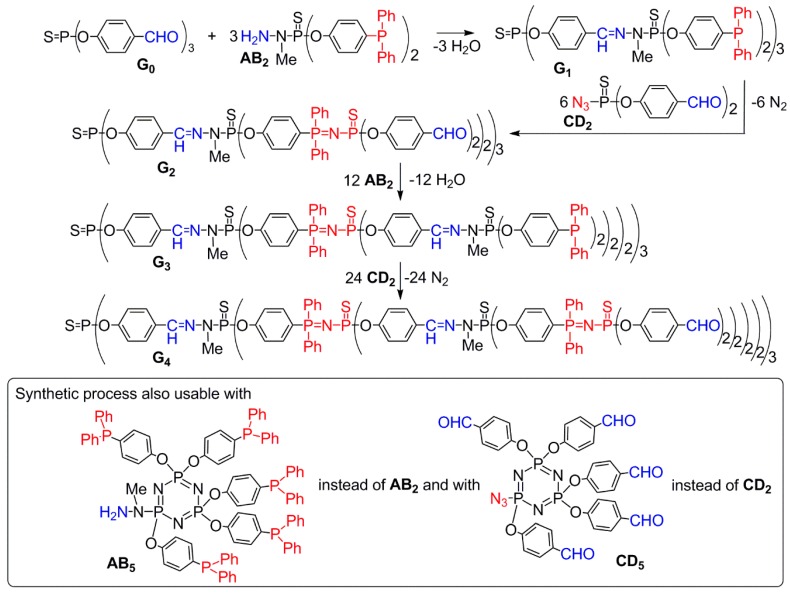
Synthesis of layered dendrimers using two branched orthogonal monomers of type AB_2_ and CD_2_, and structure of branched orthogonal monomers of type AB_5_ and CD_5_, usable in the same process.

“Layer-block” dendrimers [93] are constituted of layers of different composition, and are reminiscent to the “onion peel” dendrimers shown in the previous section. We were the first to introduce the idea of a regular alternation of “layer-blocks” for the rapid synthesis of dendrimers, using two types of branched monomers constituted of two pairs of complementary functions able to react spontaneously without any activating agent. The chosen reactions were condensation reactions between phosphorhydrazides and aldehydes on one side (reactions also used for the synthesis of classical phosphorhydrazone dendrimers, as shown in Scheme 1), and the Staudinger reactions between phosphines and azides on the other side. Two types of AB_2_ monomers, one with azide and two aldehyde functions, the other one with hydrazine and two phosphine functions were specifically designed. The step-by-step use of these branched monomers in alternation starting from (S)P(OC_6_H_4_CHO)_3_ as core has been carried out up to the fourth generation dendrimer shown in Figure 13. All reactions were quantitative, with only water and nitrogen as by-products [94]. Such synthetic process has been applied to other AB_2_ monomers, having an azide and two hydrazines for one of them, an aldehyde and two phosphines for the other one, and using a triphosphine as core. This method also afforded layered dendrimers with an alternation of P=N-P=S and hydrazine functions [95]. Another development of this process consisted in using AB_5_ branched monomers (Figure 13) instead of the AB_2_ monomers, either alone, or in alternation with AB_2_ monomers. This methodology with AB_5_ branched monomers permitted the preparation of a dendrimer of generation 3 bearing 750 phosphine terminal groups in only three steps, starting from a hexaaldehyde core [96]. In comparison, by the classical method of synthesis of phosphorhydrazone dendrimers shown in Scheme 1, only 24 terminal Cl functions are obtained after three steps, starting from the same core.

The same concept was developed further with the synthesis of an AB_2_ monomer constituted of an azide and two protected phosphines. In this case, a single monomer is necessary, as the deprotection of the phosphine terminal groups of the dendrimer at each step enables the reaction with the AB_2_ protected monomer. Dendrimers constituted of P=N-P=S linkages at all layers were built up to the fifth generation [97]. Hyperbranched polymers with a high degree of branching (0.83–0.85), also constituted of P=N-P=S linkages, were synthesized in one-step by the deprotection of the monomer. A linear polymer was synthesized in the same way using a monophosphine [98]. Comparison of the properties of the dendrimers and of the hyperbranched polymers displayed large differences in the polydispersity index as expected (PDI between 1.16 and 8.1, with Mw ranging from 3600 to 36,700 for the hyperbranched polymer; PDI 1.029 for the fifth generation dendrimer with Mw = 60,200). Furthermore, the variation of the intrinsic viscosity with Mw is very different: a bell-shaped curve for the dendrimers, with a maximum for the third generation, contrarily to an almost constant value for the hyperbranched polymers [94]. Another type of diphosphine has been used also for the synthesis of dendrimers including P=N-P=S linkages at all layers [99].

### 3.3. Reactivity of the Internal Functions (Case G)

Almost all examples of reactivity inside the structure of phosphorus dendrimers are based on P=N-P=S linkages. Indeed, the P=N-P=S linkage has a high electron density on sulfur, which induces regiospecific reactions on this atom, whereas the sulfur atoms pertaining to other P=S groups of the dendrimers do not react [100]. The early examples of internal reactions have been reviewed in details a long time ago [101], thus only the most important early examples will be given here, together with more recent results. The first example concerned the alkylation with methyl triflate of P=N-P=S linkages located at the core, issued from Staudinger reactions with Ph_2_P(CH_2_)_6_PPh_2_, followed by construction of phosphorhydrazone dendrimers up to generation 4. Analogous dendrimers were built up to generation 7, having three layers of P=N-P=S linkages, close to the core, at the level of the fifth generation, and on the surface. It was demonstrated by ^31^P-NMR spectroscopy that the alkylations occurred on all the sulfur atoms included in P=N-P=S linkages, and exclusively on them [102]. Functional triflates such as allyl triflate and propargyl triflates were also used for alkylating these linkages [103].

**Figure 14:**
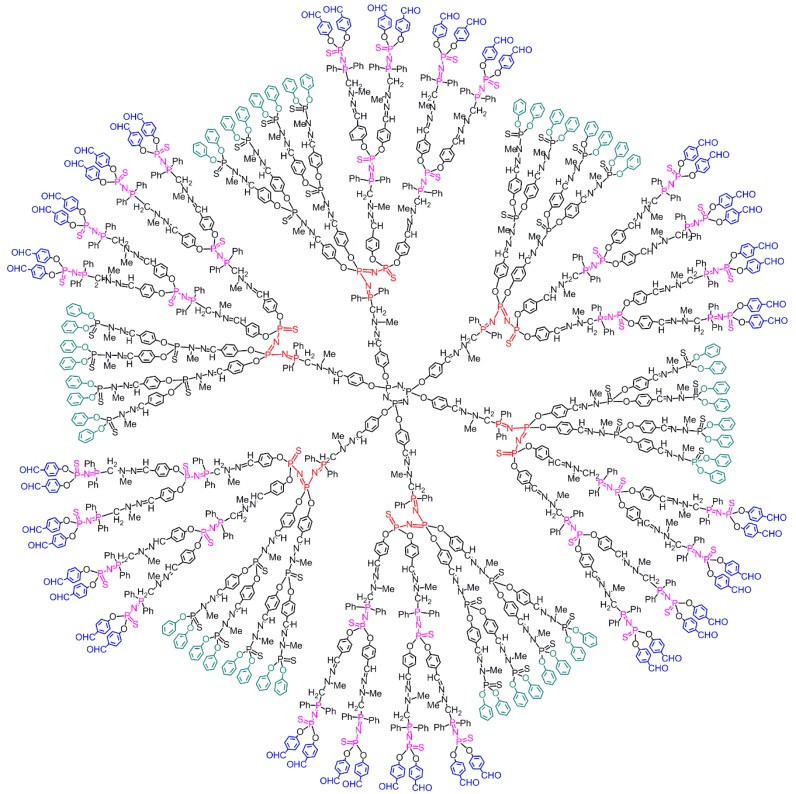
Dendritic structure with two types of functions disposed as patches on the surface of the dendrimer.

Alkylation of sulfur induces a weakening of the P=S bond, which can be easily cleaved using a nucleophilic phosphine, such as P(NMe_2_)_3_, affording phosphine groups inside the structure of the dendrimers. These phosphines can be alkylated, for instance with allyl iodide, or reacted with various azides via Staudinger reactions, affording P=N-P=N linkages and various other functional groups such as isothiocyanate, primary amine, azide, or crown ether [104]. The most important reactions concerned the Staudinger reaction with the azido dialdehyde CD_2_ shown in Figure 13, which created P=N-P=N-P=S linkages inside the structure. Indeed, the growing of new branches can be carried out from the internal aldehydes, affording very original dendritic structures, with two types of functions disposed as patches on the surface of the dendrimers (Figure 14) [105]. Analogous compounds were obtained by reaction of second generation dendrons having a hydrazine as core to the internal aldehydes of the dendrimer, showing that there is enough space inside the dendrimer to accommodate even bulky groups [62]. Zwitterionic metallocene complexes were also obtained as internal functions, by reaction of 2-phosphino-1-zirconaindene with the internal aldehydes [106].

An azide functionalized by two pyrene groups was also grafted via Staudinger reactions inside the dendrimers. Study by steady-state fluorescence spectroscopy and decays of excitation of this specially-labelled dendrimer dissolved in acetonitrile, diglyme, 1,4-dioxane, triethylene glycol, and cyclohexanol revealed that many solvent molecules penetrate inside the dendrimers, and that movements of internally located pyrene labels were not reduced by interactions with the dendrimer structure. However, for the most viscous solvent, triethylene glycol, an extensive reduction of pyrene label mobility was noticed [107].

A totally different way for the internal functionalization of dendrimers was based on the specific reactivity of amines and hydrazides with only one Cl on each P(S)Cl_2_ or P(O)Cl_2_ terminal group of phosphorus dendrimers. The grafting of hydroxybenzaldehyde on the remaining P–Cl functions, followed by the subsequent growing of the dendrimer from the aldehyde afforded dendrimers with internal functions at one generation. This type of specific reaction on P(S)Cl_2_ is one of the easiest way to introduce two types of terminal functions on the surface of the dendrimer, as will be emphasized later (Section 4.2). In the case of the internal functionalization, allyl and pyrene derivatives have been grafted in this way inside the dendrimers during their growing [108].

## 4. Dendritic Structures with Two Types of Terminal Functions in Close Proximity

We have already displayed the cases of dendritic structures having two types of functional groups either on two different wedges (case D in Figure 1) or as patches (some examples of case G). In this section, we will display examples in which two (or more) functions are close along the surface, namely cases H and I in Figure 1.

### 4.1. Dendritic Structures Having Sequentially Two Types of Terminal Functions (Case H)

**Figure 15:**
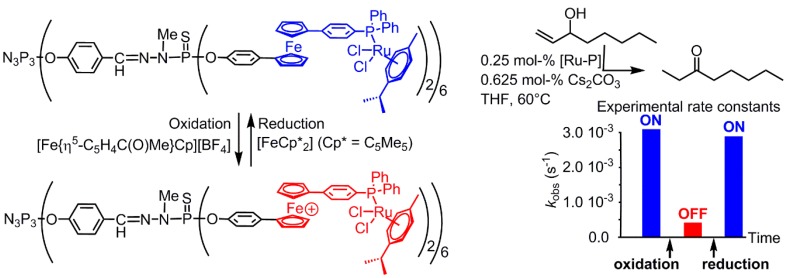
Isomerization of 1-octen-3-ol carried out with a catalytic amount of ferrocenylphosphino-Ru complex of dendrimer. Switch ON/OFF/ON experiments and influence on the catalytic activity.

The very first examples of phosphorus dendrimers having sequentially two types of terminal functions were obtained from both aldehyde and P(S)Cl_2_ terminal functions. Condensations with aldehydes afforded imines and crown ether functions, whereas Wittig reactions afforded vinyl ketones or vinyl nitriles. Reaction of functionalized amines with P(S)Cl_2_ functions afforded secondary amines and allyl or propargyl functions [109]. Other examples concerned ferrocenylaldehydes, which were used as building blocks for introducing ferrocenes inside the structure, by reaction of the aldehydes, as already shown in Figure 10 [76,77,78,110]. Other functions can be linked to the ferrocene, in particular a phosphine, usable for the complexation of ruthenium. These compounds display catalytic properties, in particular for the isomerization of allylic alcohols to ketones [111]. The presence of the ferrocene can greatly influence the catalytic outcome of these catalytic dendrimers. Indeed, the ferrocene can be oxidized to ferrocenium (electrochemically or chemically). Such oxidation greatly modify the electronic properties of the catalytic entities (ruthenium complex of phosphine), and thus the rate of catalysis. Redox-switchable catalysis (RSC) is a field of growing importance for which oxidation and reduction influence the electron-donating ability of a ligand and thus result in altered activity or selectivity of the catalyst [112], but this concept was never applied to dendrimers before our work. In the case of the isomerization of allylic alcohols, in the oxidation step, addition of 1 equiv. of [Fe{η_5_-C_5_H_4_C(O)Me}Cp][BF_4_] per catalytic function induced a markedly reduced rate. After reduction with [FeCp*_2_] (Cp* = C_5_Me_5_), full conversion was observed with a degree of activity similar to that of the original catalyst before oxidation, as shown in Figure 15 [113].

### 4.2. Dendritic Structures with Two Types of Functions on Each Terminal Branching Point (Case I)

**Figure 16:**
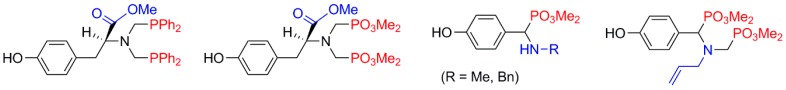
Examples of bifunctional phenols used for the grafting to P(S)Cl_2_ terminal functions of dendrimers.

In many cases, a stochastic functionalization on the surface of the dendrimer is carried out to have multiple types of functions [114]. This approach is paradoxical, as the synthesis of dendrimers aims at obtaining pure compounds, whereas the stochastic functionalization in the last step affords a mixture of compounds, which in particular can induce inconsistencies in biological responses [115]. Thus, it is highly desirable to develop tools for the precise diversification of the terminal groups of dendrimers. The simplest way consists in using a compound that already possesses two or more functions, in addition to the function needed for the grafting to the dendrimer. Several examples of bifunctional phenols are shown in Figure 16. 

The diphosphine has been grafted to dendrimers with P(S)Cl_2_ terminal functions up to generation 3, and the corresponding palladium complexes have been tested in Sonogashira and Heck couplings [116]. The phenols bearing one methylphosphonate and a secondary amine, or two methylphosphonates and an ester [38] or a vinyl group [117] have been grafted to the first generation dendrimers. After deprotection of the methylphosphonates to phosphonic salts, the biological properties of these dendrimers have been tested, in particular for the activation of monocytes. 

**Figure 17:**
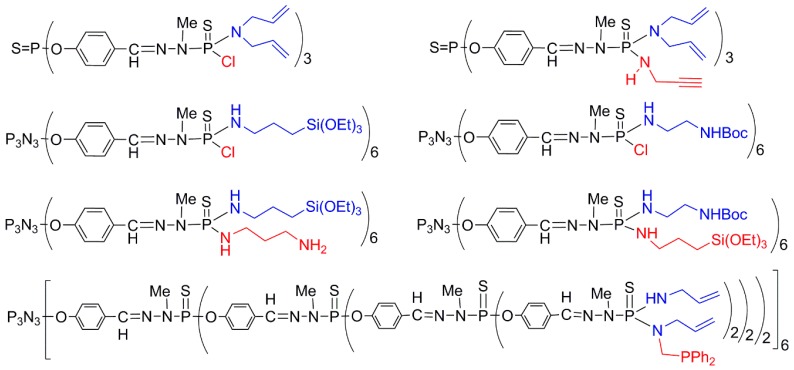
Examples of selective and sequential functionalization of each Cl on P(S)Cl_2_ functions, or on a single NH function (**bottom**).

We have studied for a long time the original possibility of differentiating the reactivity of each Cl on the terminal P(S)Cl_2_ functions. This process is particularly useful for the reaction of primary or secondary amines. In a first approach, allyl and propargyl groups were grafted on the surface of dendrimers up to generation 4 (see Figure 17 for the first generation) [118]. More recently, this approach has been applied for obtaining free or protected primary amines, together with triethoxysilanes as terminal groups (Figure 17), for the grafting to silica [33] for trapping CO_2_ [119]. The specific functionalization has been observed also after the reaction of primary amines with P(S)Cl_2_ terminal groups; indeed, only one NH reacted with Ph_2_PCH_2_OH, even when used in large excess. This work was carried out up to the seventh generation; the fourth generation is shown in Figure 17 [120].

**Figure 18:**
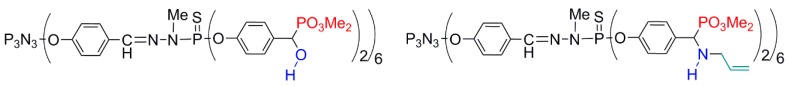
Results of the addition of dimethylphosphonate on terminal aldehydes (**left**) or imines (**right**).

Other examples of post-functionalization leading to two (or three) types of terminal groups include the addition of the phosphonate HPO_3_Me_3_ on aldehydes, affording a phosphonate and an alcohol. This dendrimer was tested for the activation of monocytes [38], but also for obtaining catanionic (saline, containing both cationic and anionic centers) multivalent analogs of GalCer (galactosylceramide) by interaction of this dendrimer with N-hexadecylaminolactitol moieties [121]. These types of catanionic systems proved to be good HIV-1 inhibitors [122]. Addition of phosphonate HPO_3_Me_3_ can be carried out also on imine terminal functions, affording in this case three types of terminal functions, phosphonates, secondary amines, and alkyl groups (Figure 18) [117].

## 5. Conclusions

Phosphorus-containing dendrimers constitute a special class of dendrimers, which possess advantageous properties related to the versatility of their synthesis and the multiple possibilities and modularity of their functionalization. One important property is the large variety of possibilities offered by these dendrimers to elaborate bifunctional structures, that is to say dendritic structures possessing two (or more) types of functional groups. Indeed, these dendrimers offer the possibility to have functional groups at the core (or close to it), within the branches, but also two types of precisely organized terminal functions. Thanks to these special features, phosphorus-containing dendrimers have already been used as reusable and efficient catalysts, for the elaboration of nanomaterials, as fluorescent agents for bio-imaging, and as drugs by themselves, to name as a few uses. All these properties pave the way for the search of other properties, which will surely benefit from the tools already elaborated for having two types of functional groups, in precise positions of dendritic structures.

## Abbreviations

The following abbreviations are used in this manuscript:

A549 cells	Adenocarcinomic human alveolar basal epithelial cells
3-APDMES	3-AminoPropyl DiMethylEthoxySilane
B14 cells	Chinese hamster fibroblasts
Boc	tert-ButylOxyCarbonyl
CD	Circular Dichroism
Cp	CycloPentadienyl
Cp*	C_5_Me_5_
Cyclam	1,4,8,11-tetraazacyclotetradecane
dansyl	5-(DimethylAmino)Naphthalene-1-SulfonYL
dba	DiBenzylidene Acetone
DFT	Density Functional Theory
DNA	DeoxyriboNucleic Acid
EGFP	Enhanced Green Fluorescent Protein
Felbinac	biphenylylacetic acid
GalCer	GalactosylCeramide
HEK 293 cells	Human Embryonic Kidney cells
HeLa cells	cervical cancer cells taken from the patient Henrietta Lacks
HIV	Human Immunodeficiency Virus
HSA	Human Serum Albumin
HUVEC cells	Human Umbilical Vein Endothelial Cells
IR	Infra-Red
LbL	Layer-by-Layer
MTT	diMethyl Thiazolyl diphenyl Tetrazolium salt
Mw	weight average Molecular Weight
N2a cells	fast-growing mouse Neuroblastoma cell line
NK cells	Natural Killer cells
NMR	Nuclear Magnetic Resonance
PAMAM	PolyAMidoAMine
PDI	PolyDispersity Index
PEG	PolyEthyleneGlycol
Rh	hydrodynamic Radius
ROS	Reactive Oxygen Species
RSC	Redox-Switchable Catalysis
TEOS	TetraEthOxySilane)
TFA	TriFluoroacetic Acid
THF	TetraHydroFuran
TPA	Two-Photon Absorption
Triflate	Trifluoromethanesulfonate
UV	Ultra-Violet
